# Distinct microbiome profiles and biofilms in *Leishmania donovani*-driven cutaneous leishmaniasis wounds

**DOI:** 10.1038/s41598-021-02388-8

**Published:** 2021-11-30

**Authors:** T. D. Jayasena Kaluarachchi, Paul M. Campbell, Rajitha Wickremasinghe, Shalindra Ranasinghe, Renu Wickremasinghe, Surangi Yasawardene, Hiromel De Silva, Chandrani Menike, M. C. K. Jayarathne, Subodha Jayathilake, Ayomi Dilhari, Andrew J. McBain, Manjula M. Weerasekera

**Affiliations:** 1 Department of Parasitology, Faculty of Medical Sciences, University of Sri Jayewardenepura, Gangodawila, Nugegoda, Sri Lanka; 2 Division of Pharmacy and Optometry, School of Health Sciences, Faculty of Biology, Medicine and Health, The University of Manchester, Manchester, UK; 3 Department of Public Health, Faculty of Medicine, University of Kelaniya, Kelaniya, Sri Lanka; 4 Department of Anatomy, Faculty of Medical Sciences, University of Sri Jayewardenepura, Gangodawila, Nugegoda, Sri Lanka; 5 Dermatology Unit, Base Hospital, Tangalle, Sri Lanka; 6 Department of Family Medicine, Faculty of Medical Sciences, University of Sri Jayewardenepura, Gangodawila, Nugegoda, Sri Lanka; 7 Department of Pathology, Faculty of Medical Sciences, University of Sri Jayewardenepura, Gangodawila, Nugegoda, Sri Lanka; 8 Department of Basic Sciences, Faculty of Allied Health Sciences, University of Sri Jayewardenepura, Gangodawila, Nugegoda, Sri Lanka; 9 Department of Microbiology, Faculty of Medical Sciences, University of Sri Jayewardenepura, Gangodawila, Nugegoda, Sri Lanka

**Keywords:** Biofilms, Microbial communities, Parasitology

## Abstract

The endemic strain of *Leishmania donovani* in Sri Lanka causes cutaneous leishmaniasis (CL) rather than more common visceral form. We have visualized biofilms and profiled the microbiome of lesions and unaffected skin in thirty-nine CL patients. Twenty-four lesions (61.5%) were biofilm-positive according to fluorescence in situ hybridization. Biopsies of biofilm-positive lesions were dominated by *Pseudomonas*, class *Bacilli* and *Enterobacteriaceae* and distinguished by significantly lower community evenness. Higher relative abundance of a class *Bacilli* OTU was detected in wound swabs versus contralateral skin. Wound swabs and biopsies had significantly distinct microbiome profiles and lower diversity compared to unaffected skin. Greater abundances of potentially pathogenic organisms were observed in wet ulcers, lesions with high parasite loads and large wounds. In summary, more than half of *L. donovani* associated CL wounds harboured biofilms and the wounds exhibited a distinct, less diverse, microbiome than unaffected skin.

## Introduction

*Leishmania donovani*-driven cutaneous leishmaniasis is endemic to Sri Lanka. *L. donovani*, which usually causes visceral leishmaniasis, causes cutaneous leishmaniasis in Sri Lanka, parts of India, Sudan, Lebanon, and Turkey^1^. Cutaneous leishmaniasis can lead to disfiguring ulcers, and the lesions are chronic, taking months to years to completely heal (with or without anti-parasitic treatment)^2^. Some of the severe forms of cutaneous leishmaniasis occur due to host factors, such as elevated Type 2 cytokines and imbalances between tissue inhibitors of metalloproteinase and matrix metalloproteinase^3,4^. Gimblet et al., (2017), proposed that the imbalance of the lesional skin microbiome in cutaneous leishmaniasis wounds caused by *L. major* plays a major role in altering disease severity and duration^5^. To date, however, relatively few studies have been published on cutaneous leishmaniasis wound microbiome and little information is available on the microbiome of lesions caused by *L. donovani*.

Patients with chronic ulcerated skin lesions are prone to develop bacterial biofilms which can function as barriers to antibiotic treatment^6^, reduce fibroblast deposition and increase inflammation resulting in impaired wound healing^7^. Biofilms are also known to interact with protozoans in nature^8^. Even though cutaneous leishmaniasis wounds are largely symptomless, a “burning sensation” is common, purportedly reflecting an inflammatory reaction to biofilms^9^. In managing chronic wounds, improved therapeutic modalities could potentially be developed by targeting biofilms^10^ although the current understanding of the involvement of biofilms in cutaneous leishmaniasis wounds is incomplete.

We hypothesized that cutaneous leishmaniasis wounds caused by *L. donovani* would be associated with distinct microbiome profiles compared to unaffected skin, and that microbial biofilms would form in a proportion of cutaneous leishmaniasis wounds with possible etiological implications. We additionally hypothesized that the microbiome in cutaneous leishmaniasis wounds would differ with lesion size, parasite load and type of lesion.

Thus, we characterized the microbiome of cutaneous leishmaniasis wounds caused by *L. donovani* comparing it with adjacent and contralateral skin. Also, we visualized and profiled bacterial biofilms in these wounds. The information presented in this manuscript would be useful to gain a better understanding of the cutaneous leishmaniasis microbiome to facilitate future research on wound management.

## Results

Thirty-nine patients were confirmed as cutaneous leishmaniasis positive by PCR. The group included 21 males and 18 females. The mean age was 46.2 years. The investigation included ulcers (wet ulcers n = 10, dry ulcers n = 10), ulcerated nodules (n = 13), ulcerated papules (n = 4) and ulcerated plaques (n = 2). The mean duration of those wounds at the time of presentation was 16.4 weeks.

### The microbiome of cutaneous leishmaniasis wounds caused by *Leishmania donovani*

DNA was sequenced from 157 samples. Four sites were sampled from 39 patients, including wound biopsies, wound swabs, contralateral skin swabs and adjacent skin swabs. A negative control of purified water was included, which had only 5 reads following quality filtering (raw reads n = 77) and was negligible compared to the high read numbers of the actual samples. This control was, therefore, not subtracted from the analysis.

There were 13,175,234 raw reads of which 3,686,663 were from contralateral skin swabs, 3,462,115 were from adjacent skin swabs, 3,906,256 were from wound swabs and 2,120,200 were from wound biopsies. There were 7,342,254 quality-filtered sequences with an average of 46,765.95 reads per sample. Each read was approximately 220 bp in size. The reads were allocated to 17,032 operational taxonomic units (OTUs). The mean frequency per OTU was 431.08.

The taxonomic composition of the data included 51 phyla, 151 classes, 302 orders, 551 families and 1168 genera.

### Diversity analysis of the microbiome data of cutaneous leishmaniasis wounds caused by *Leishmania donovani*

During diversity analysis, data were rarefied to an even depth of 20,000. Thirteen wound biopsy samples and one adjacent skin swab sample, which had a low amount of reads were below this threshold and, hence, not included in diversity analyses.

**Figure 1:**
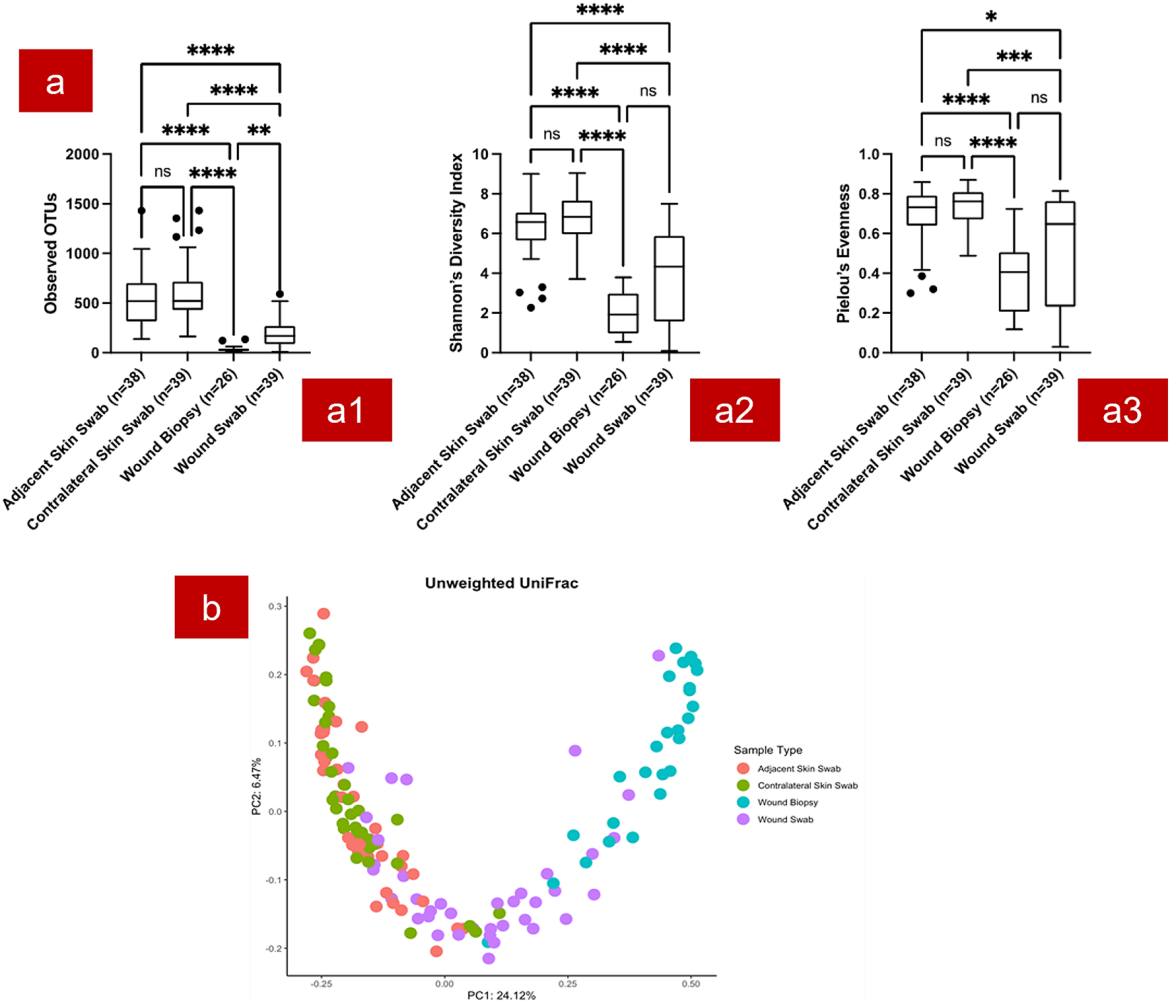
Diversity analysis results of different sample types. (**a**) Bacterial diversity based on sample location. Observed OTUs (a1); Shannon–Wiener diversity index (a2); Pielou’s index (a3); (**b**) Unweighted Unifraction distance matrix-based ordination (β diversity). Statistical significance of the difference between each sample type is denoted with asterisks. *p ≤ 0.05; **p ≤ 0.01; ***p ≤ 0.001; ****p ≤ 0.0001; ns: *p* > 0.05.

Alpha-diversity parameters were evaluated for sample richness (Observed OTUs), diversity (Shannon–Weaver diversity) and community evenness (Pielou’s index) (Fig. 1a). Statistical significance between each sample type was assessed using the pairwise Kruskal–Wallis test. We found no statistically significant differences in sample richness, Shannon diversity or community evenness between contralateral skin swab and adjacent skin swab samples (*p* > 0.99). Wound swabs and wound biopsies were significantly different to the “healthy” skin swabs (contralateral and adjacent skin swabs, *p* < 0.001). There was no significant difference found between wound swab and biopsy in terms of diversity and evenness. However, wound swabs had a significantly higher richness (Observed OTUs) compared to wound biopsies.

Unweighted unifraction distance matrix (Fig. 1b) showed clear separation of wound biopsy samples. Wound swabs and biopsies had a significant dissimilarity of bacterial composition compared to contralateral skin swabs and adjacent skin swabs (pairwise Permanova test p value < 0.001 for all combinations). The healthy skin swabs had similar bacterial compositions (pairwise Permanova test p value = 0.926).

### Relative abundance of the OTUs associated with the cutaneous leishmaniasis microbiome

**Figure 2:**
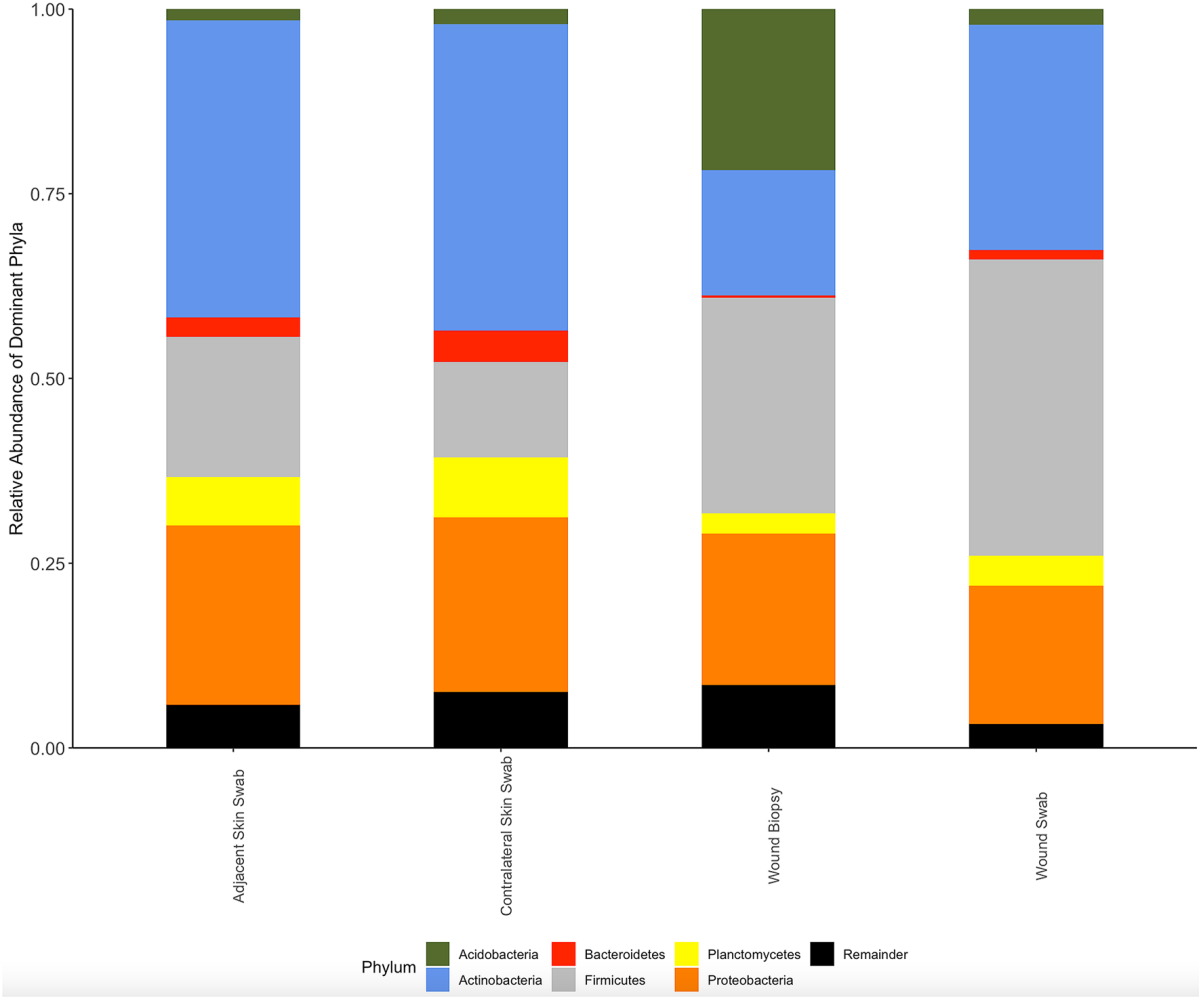
Relative abundance of the phyla in adjacent skin swabs, contralateral skin swabs, wound biopsies, and wound swabs. (Figure generated by R Studio version 1.3.1093 available at http://www.rstudio.com/. Using R version 4.0.3 available at https://www.R-project.org/).

The top 5 phyla according to the relative abundance in contralateral skin swabs were *Actinobacteria* (41.55%), *Proteobacteria* (23.71%), *Firmicutes* (12.92%), *Planctomycetes* (8.04%) and *Bacteroidetes* (4.23%). The same 5 phyla were dominant in the adjacent skin swabs. In the wound swabs, *Firmicutes* were dominant (40.07%) followed by *Actinobacteria* (30.48%), *Proteobacteria* (18.70%), *Planctomycetes* (4.08%) and *Acidobacteria* (2.16%). In wound biopsies, the top 5 phyla were *Firmicutes* (29.18%), *Acidobacteria* (21.81%), *Proteobacteria* (20.51%), *Actinobacteria* (17.00%) and *Deinococcus–Thermus* (4.42%). Phylum *Fusobacteria* was generally low in all sample types and relatively absent in wound biopsies. In contralateral skin, adjacent skin and wound swabs, phylum *Euryarchaeota* and *Parvarchaeota* (Under Kingdom *Archaea*), were detected in very low relative abundances (< 0.1%) and they were frequently absent in wound biopsy samples. Figure 2 shows the relative abundance of the dominant phyla in which the low abundant phyla (< 1% of relative abundance) are grouped as “Remainder”.

**Figure 3:**
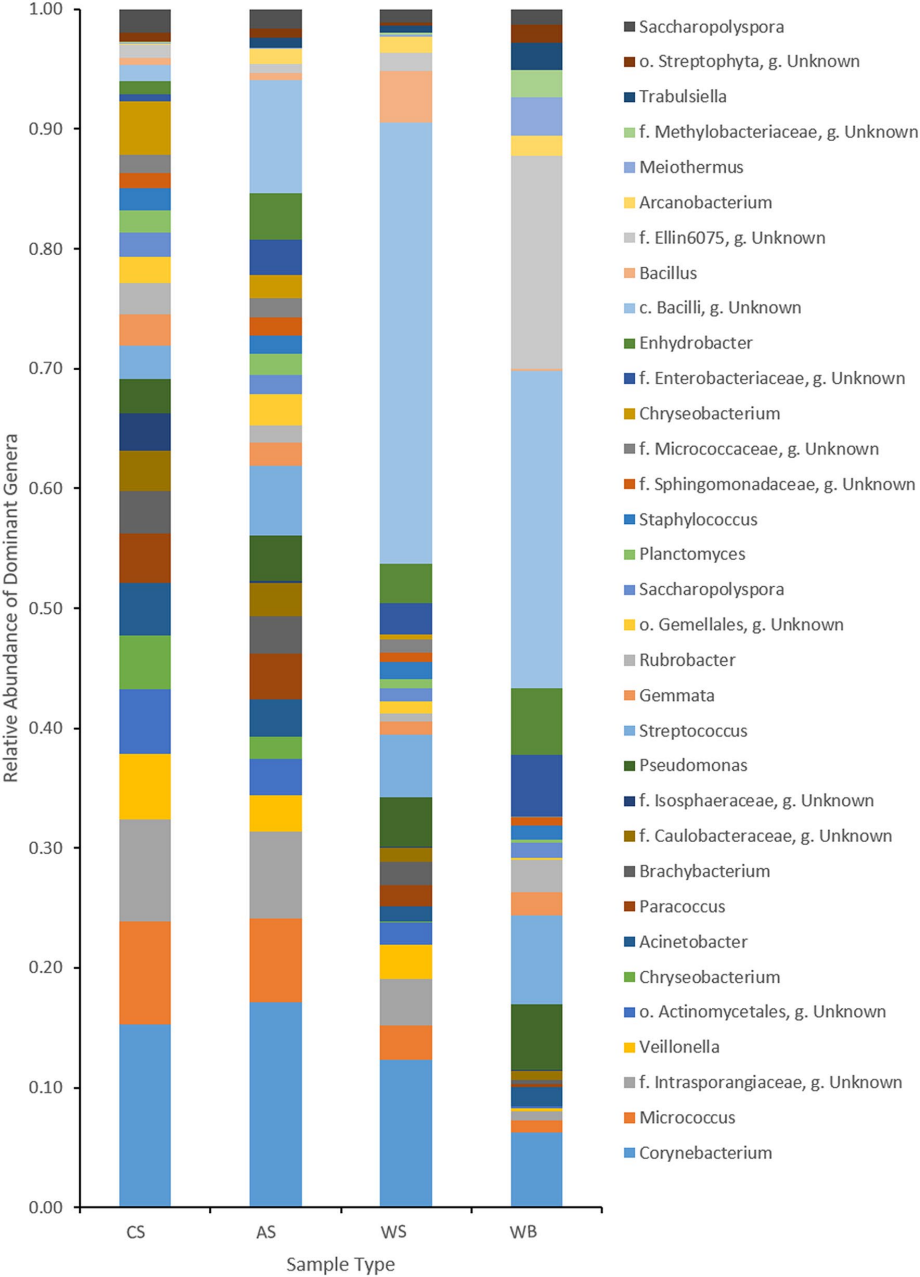
Stacked bar plots with dominant genera (relative abundance > 0.5%) in contralateral skin swabs (CS), adjacent skin swabs (AS), wound swabs (WS) and wound biopsies (WB). f: Family; o: Order; g: Genus. (Figure generated by Microsoft Excel 2013).

DESeq2 analysis showed a significant difference in the relative abundance of 18 OTUs including OTUs belonging to the genera *Actinobacter*, *Planctomyces*, *Nocardioides*, *Staphylococcus*, *Balneimonas* and *Streptococcus* between contralateral skin swabs and wound swabs. An OTU belonging to class *Bacilli* had a higher relative abundance (statistically significant p.adj < 0.05, 20.51%) in wound swabs than contralateral skin swabs (0.1%). Several OTUs (n = 5), including *Cloacibacterium*, were also found to be significantly different in wound swabs compared to adjacent skin swabs (Supplementary Table 1). An OTU belonging to family *Ellin6075* was significantly higher in relative abundance in wound swabs (1.24%) than adjacent skin swabs (0.07%). There were 50 OTUs significantly different in relative abundance in wound biopsies compared to wound swabs. In wound biopsies, OTUs of family *Ellin6075*, *Streptococcus*, *Enhydrobacter*, *Rubrobacter*, *Meiothermus* were significantly higher in relative abundance compared to wound swabs. *Pseudomonas* was in higher relative abundance in both wound swabs (3.34%) and wound biopsies (4.90%) than healthy skin (Fig. 3, Supplementary Table 1). Genus *Mycobacterium* was present in a relative abundance of 0.06%.

### Microbiome profile comparison of; parasite load, lesion size and type of cutaneous leishmaniasis lesions

DESeq2 analysis showed that the relative abundance of an OTU of *Trabulsiella* was significantly higher in lesions with high parasite loads compared to lesions with low parasite loads (p.adj < 0.05) and OTUs belonging to family *Ellin6075* and *Arcanobacterium* spp. were significantly higher in small (< 2 cm) cutaneous leishmaniasis lesions (p.adj < 0.05) whereas OTUs of *Corynebacterium* and class *Bacilli* were significantly higher in large (> 2 cm) lesions (p.adj < 0.05).

Alpha and beta diversity assessments of the wound biopsy samples from different types of cutaneous leishmaniasis lesions indicated that there was no significant dissimilarity in terms of microbial composition (*p* > 0.05 for the overall Kruskal–Wallis test and the following pairwise Permanova tests for all combinations). DESeq2 analysis of different lesion types revealed no OTUs significantly different between dry and wet cutaneous leishmaniasis wounds. However, there were 3 OTUs belonging to the genus *Enhydrobacter*, *Bradyrhizobium* and *Actinomyces* which were significantly higher in relative abundance in ulcerative nodules compared to wet ulcers (p.adj < 0.05). Also, an OTU belonging to *Corynebacterium* was significantly higher in relative abundance in ulcerative nodules compared to dry ulcers (p.adj < 0.05) and an OTU belonging to *Enhydrobacter* was significantly higher in relative abundance in dry ulcers than ulcerative nodules (p.adj < 0.05). Two OTUs belonging to genus *Corynebacterium* and *Micrococcus* were significantly higher in relative abundance in wet ulcers compared to ulcerated papules (p.adj < 0.05), in which these two OTUs were relatively absent.

### Visualizing bacterial biofilms in cutaneous leishmaniasis wounds caused by *Leishmania donovani*

A pig-skin model was used as a positive control for fluorescence in situ hybridization assay on formalin-fixed paraffin-embedded tissue (FFPE-FISH) and Gram staining of formalin-fixed paraffin-embedded tissue (FFPE-gram) and scanning electron microscopy (SEM) imaging. For the FFPE-FISH section, a CY3 tagged EUB probe was used to stain bacteria red, Concavalin A conjugated Alexa Fluor 488 marked the extracellular polymeric substances in green and DAPI marked the nuclei in blue. For the FFPE-Gram section, bacteria were Gram-stained, and the extracellular polymeric substances was stained orange by Safranin. SEM imaging allowed visualization of both cocci and bacilli (Supplementary Fig. 1).

Wound biopsies of the 39 cutaneous leishmaniases confirmed (by PCR) lesions of patients were visualized for bacterial biofilms. Of them, 24 (61.5%) lesions were positive for biofilms by FFPE-FISH. By SEM, 23 out of the 39 lesions (59.0%) were biofilm positive. One sample which was biofilm positive by FFPE-FISH was found to be biofilm negative using SEM. FFPE-Gram detected 14 (35.9%) lesions as biofilm positive. Fourteen samples were confirmed as biofilm positive and 15 were biofilm negative by all three imaging techniques and 10 were positive at least by one method. The mean size of the biofilms in these cutaneous leishmaniasis lesions was 47.9 µm (SD +/− 32.9, range = 6.8–141.7). There were 2 biofilm lesions 5–10 μm, 13 lesions 11–50 μm, and 9 lesions > 50 μm in size.

FFPE-FISH, FFPE-Gram and SEM images of cutaneous leishmaniasis wounds are shown in Supplementary Fig. 2, 3a and 3b-c respectively. In some samples, identification of the bacterial red signal by FFPE-FISH was hindered by auto-fluorescent granules of the skin and the extracellular polymeric substances was less (or not) evident. In the FFPE-Gram sections, most of the biofilm positive samples had gram-positive cocci bacterial aggregates. Isolated small aggregates of bacteria were apparent in most of the slides. However, in most of the sections, demonstration of the extracellular polymeric substances and conclusion on biofilm positivity due to the small size of the aggregates was difficult. SEM images of the samples also showed coccoid cells, either in clumps, chains, or both, and the extracellular substance could be demonstrable. Different textures of the extracellular substance could be seen by SEM i.e., smooth, and thin thread-like.

#### The association of bacterial biofilms in cutaneous leishmaniasis wounds with clinico-demographic characteristics of the study cohort

The association between the presence of biofilm in CL lesions and relevant clinico-demographic variables are summarized in Supplementary Table 2.

The mean age of the group was 46.2 years (SD ± 16.2, range = 18–77). There was a significant association between biofilm formation and age (*p* = 0.031) with patients over 40 years having a higher percentage of biofilm formation. Wet lesions had a significantly higher biofilm formation as compared to dry lesions (*p* = 0.004). Symptomatic lesions had significantly higher biofilm positivity as compared to asymptomatic lesions (*p* = 0.015). All lesions with pus cell count ≥ 3 + had biofilms and all the biofilm negatives had a pus cell count of < 3 + (*p* = 0.007). Biofilm positivity was higher in males (58.3%), in upper limb lesions (62.5%) in CL lesions of < 3 months (70.8%) and in lesions with high parasite counts (62.5%) though not significantly different from the respective comparison group.

#### Profiling of the wound biopsies containing bacterial biofilms

Eight biofilm positive wound biopsy samples and five biofilm negative wound biopsy samples, which had a low amount of reads, did not meet the rarefaction threshold and were excluded from the diversity analysis.

**Figure 4:**
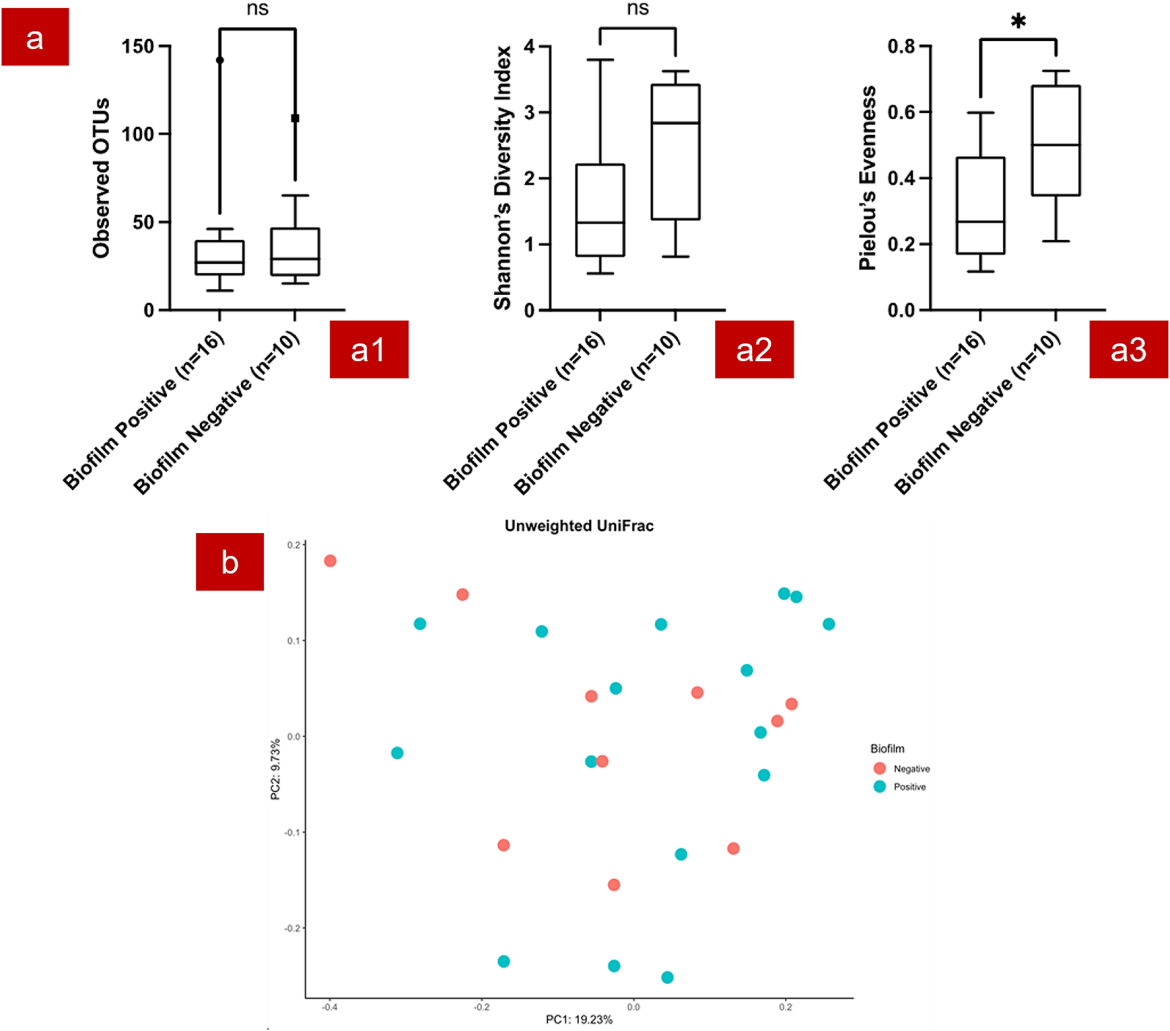
Diversity analysis results of the biofilm positive and negative wound biopsies. (**a**) Bacterial diversity based on biofilm positivity in wound biopsies. Observed OTUs (a1); Shannon–Wiener diversity index (a2); Pielou’s index (a3); (**b**) Unweighted Unifraction distance matrix based on biofilm positivity of the wound biopsies. *: statistically significant difference p ≤ 0.05; ns: no statistically significant difference *p* > 0.05.

There was no significant difference between the microbiome profiles of “biofilm positive” and “biofilm negative” cutaneous leishmaniasis wound biopsies in terms of alpha diversity except evenness (pairwise Kruskal–Wallis test p value for Pielou’s evenness was 0.023, Fig. 4a). The two groups had no significant difference in terms of beta diversity measurements as well (Pairwise Permanova test p value > 0.05, Fig. 4b).

**Figure 5:**
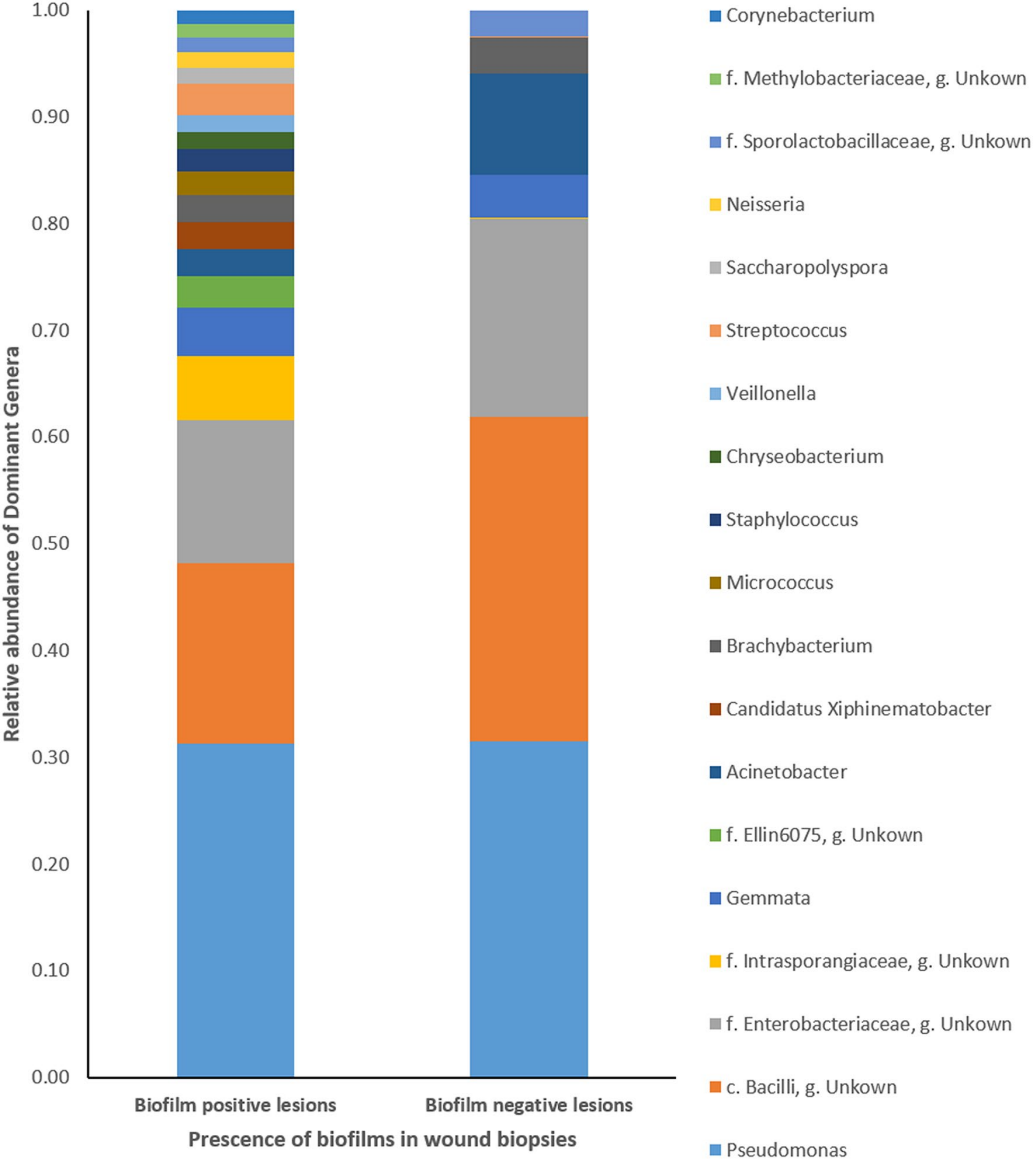
Relative abundance of genera (genera with > 1% abundance considered) in biofilm positive and negative wound biopsy samples. f: Family; o: Order; g: Genus. (Figure generated by Microsoft Excel 2013).

DESeq2 analysis showed a significant higher relative abundance of an OTU belonging to class *Bacilli* (which includes *Staphylococcus* spp. and *Streptococcus* spp.) in “biofilm positive” wound biopsies (p.adj < 0.05). In addition, almost all the genera which were of > 1% in “biofilm positive” wound biopsies were biofilm-forming bacteria, and they were in relatively higher abundance in “biofilm positive” wound biopsies compared to “biofilm negative” wound biopsies (Fig. 5).

**Figure 6:**
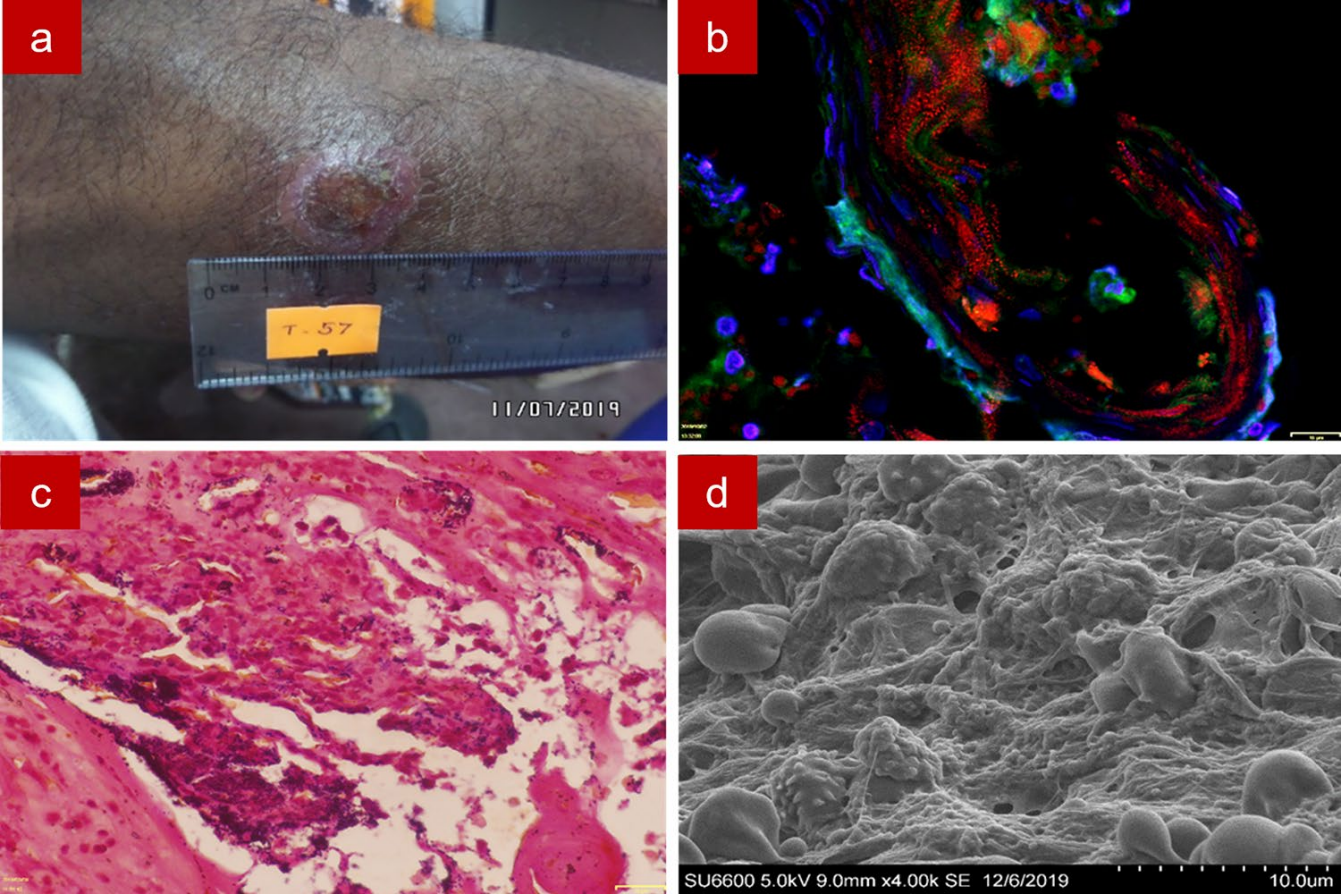
Sample 2. Image of a wet ulcer of 4 weeks duration with a yellow scab in the lower limb (**a**); Fluorescence in situ hybridization showing bacteria in red with the CY3 tagged Eu bacterial probe, extracellular polymeric substances in green with Concavalin A conjugated Alexa Fluor 488 and tissue nuclei in blue with DAPI (**b**); Gram staining showing gram-positive cocci in clumps (**c**); Scanning electron microscopy showing coccoid shaped bacteria embedded in thread-like extracellular polymeric substance (**d**).

**Figure 7:**
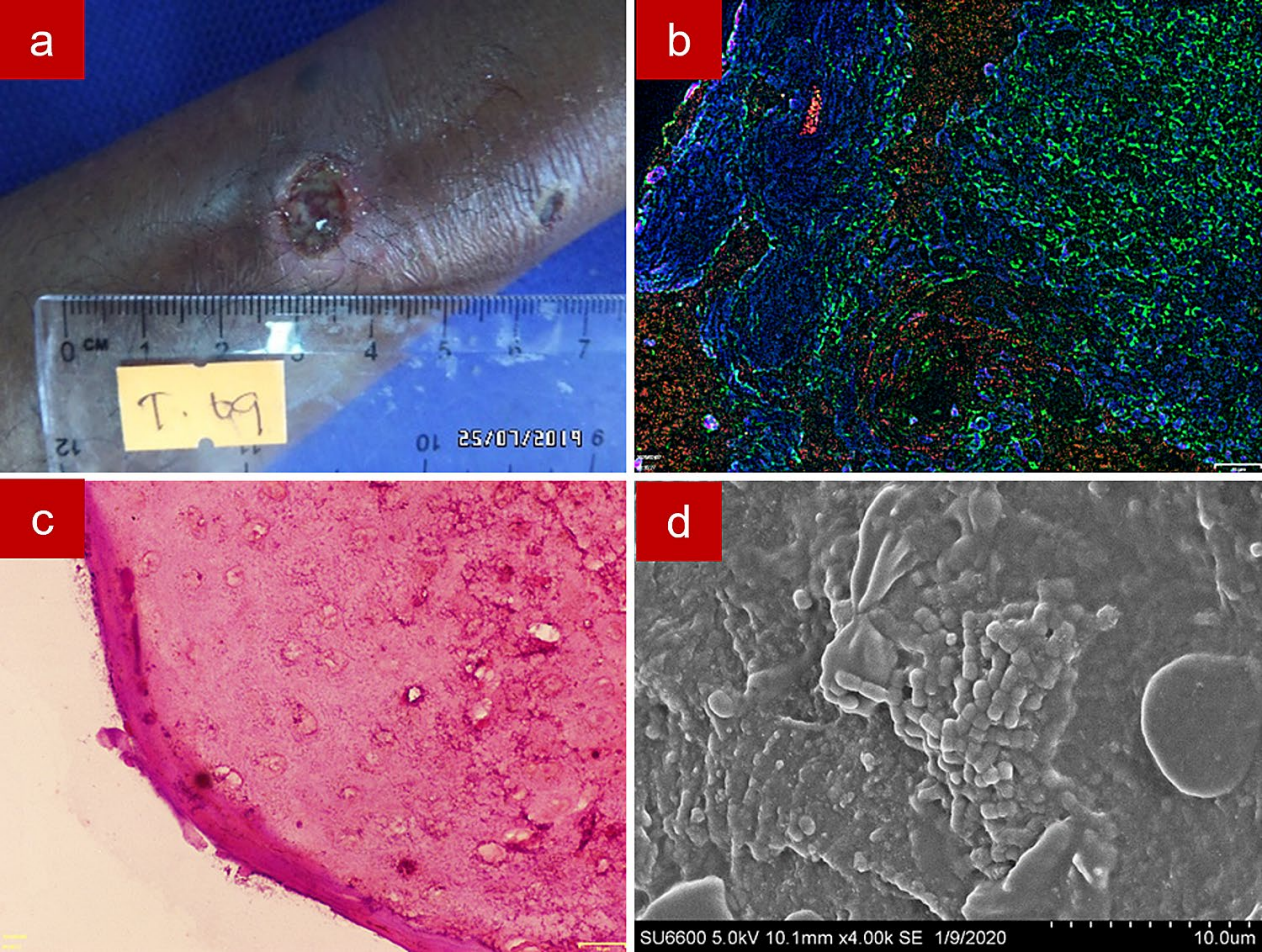
Sample 7. Image of a wet ulcer of 12 weeks duration in the lower limb (**a**); Fluorescence in situ hybridization showing bacteria in red with the CY3 tagged Eu bacterial probe, extracellular polymeric substances in green with Concavalin A conjugated Alexa Fluor 488 and tissue nuclei in blue with DAPI (**b**); Gram staining showing gram positive cocci (**c**); Scanning electron microscopy showing coccoid shaped bacteria in chains embedded in smooth extracellular polymeric substance (**d**).

**Figure 8:**
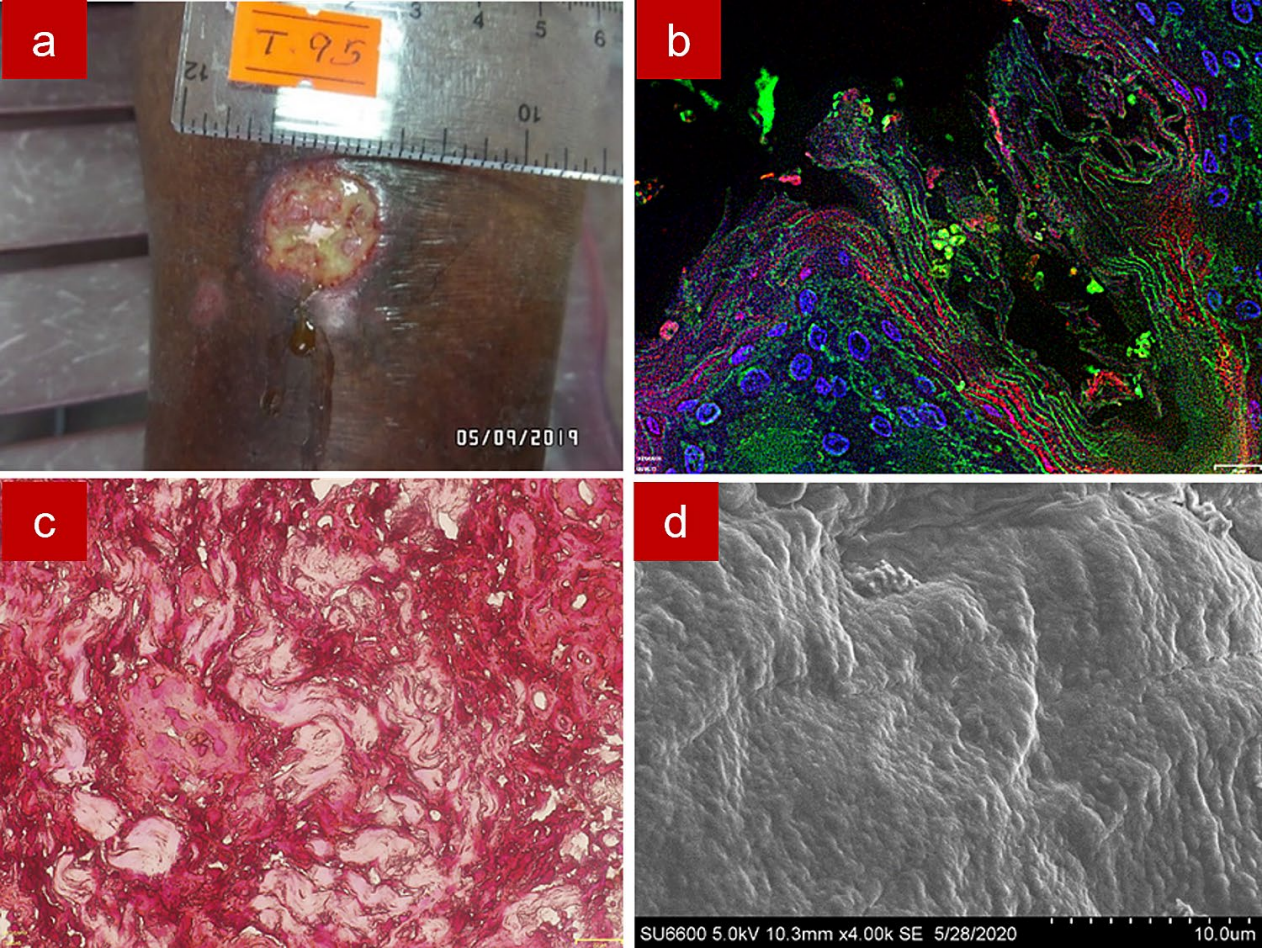
Sample 27. Image of a wet ulcer of 8 weeks duration in the lower limb with green colour pus discharge (**a**); Fluorescence in situ hybridization showing bacteria in red with the CY3 tagged Eu bacterial probe, extracellular polymeric substances in green with Concavalin A conjugated Alexa Fluor 488 and tissue nuclei in blue with DAPI (**b**); Gram staining showing gram-negative rods (**c**); Scanning electron microscopy showing bacteria in embedded in smooth extracellular polymeric substance (**d**).

Further, Figs. 6, 7 and 8 show positive bacterial biofilms (positive by all three imaging techniques), of samples 2, 7, and 27 respectively. In sample 2, class *Bacilli* was seen at a relative abundance of 89.03%. Whilst the taxonomic classification of this OTU did not resolve to the level of genus, our initial experiments run with cultures (data not shown), might allow us to infer these cocci in clumps could be *Staphylococcus* spp. belonging to order *Bacillales*. Sample 7, for example, found *Streptococcus* sp. in a relative abundance of 92.34%. In the SEM images the *Streptococc*us spp. in chains were demonstrable. Sample 27 had *Pseudomonas* sp. in a relative abundance of 94.07%.

## Discussion

We have profiled the microbiomes of cutaneous leishmaniasis lesions in the presence and absence biofilms. Cutaneous leishmaniasis lesions are chronic in nature. It has been reported that the prevalence of biofilms in non-cutaneous leishmaniasis chronic wounds is more than 78.2%^11,12^. We detected biofilms in 61.5% (24/39) of the cutaneous leishmaniasis lesions using fluorescence in situ hybridization in formalin-fixed paraffin-embedded tissue (FFPE-FISH). These cutaneous leishmaniasis lesions with biofilms were mainly associated with an OTU belonging to class *Bacilli*, a class that includes common pyogenic bacteria such as Streptococcus and Staphylococcus, and pseudomonas.

As in a previous study on diabetic wounds^13^, FFPE-FISH detected the most biofilms in cutaneous leishmaniasis lesions followed by SEM. It has been shown that Gram stain often fails to detect biofilms in lesions of less than 1-month duration^14^. In the current study, most of the cutaneous leishmaniasis lesions (59%) were of less than three months duration and FFPE-Gram failed to detect over 40% of CL biofilms that were detected by FFPE-FISH. These were wounds with biofilms of small aggregates (15/24 were < 50 µm as measured by FFPE-FISH) which may have limited detection.

All lesions with a high pus cell count of ≥ 3 + with potential infection/ inflammation had biofilms (*p* = 0.007). Gimblet et al., 2017^5^ and Salgado et al., 2016^15^ have previously suggested that changes in the cutaneous leishmaniasis microbiome might lead to increased inflammation, which could have a downstream effect on disease chronicity and severity in humans.

It has been reported that 60% of cutaneous leishmaniasis lesions caused by *Leishmania braziliensis* presented with secretions, 44% with pain and itching and 40% with burning sensation^9^. The authors concluded that secretions and burning/itching sensation of cutaneous leishmaniasis lesions are frequently associated with secondary bacterial infection and that the burning sensation may be due to biofilm formation in these lesions. Whilst we did not evaluate burning sensation in the *L. donovani*-driven cutaneous leishmaniasis wounds, we observed similar percentages of wet (59%), and symptomatic (pain and/or itching) lesions (33.3%). Since most of the biofilms in cutaneous leishmaniasis lesions were significantly seen in patients with symptomatic (pain and/or itching) (*p* = 0.015) and wet lesions (*p* = 0.004), the presence of secretions, pain and/or itching may be considered as indicators of biofilm formation.

The bacterial-parasite association in cutaneous leishmaniasis lesions is not well understood. One study showed that co-infection of *S. aureus* and *L. major* promoted *S. aureus* growth but parasite numbers remained unchanged in murine models^16^. Another in vitro study reported lysis of *L. chagasi* by *S. marcescens* SM365^17^. We did not observe any significant association between biofilm formation and *L. donovani* parasite loads. However, most of the lesions with high parasite load (62.5%) had biofilms, indicating some change in the microenvironment of these lesions facilitating biofilm formation.

In addition to the description of the biofilms in *L. donovani*-associated cutaneous leishmaniasis wounds, we assessed the microbiome of the cutaneous leishmaniasis lesions caused by *L. donovani*. Our results indicate that biopsies and wound swabs from *L. donovani*-associated sites had significantly different alpha and beta diversity profiles to swabs from healthy skin sites. Beyond this, analysis of the OTUs present in each sample type revealed key characteristics of the *L. donovani*-associated cutaneous leishmaniasis microbiome, in comparison to previously described cutaneous leishmaniasis wounds.

Salgado et. al., 2016 reported that *Firmicutes* (54.3%), *Actinobacteria* (11.7%) and *Fusobacteria* (11.6%) were the top phyla in wound swabs of cutaneous leishmaniasis lesions caused by *Leishmania braziliensis*^15^ in a Brazilian cohort. In the present study, we observed a considerably lower relative abundance of *Firmicutes* (40.07%) and higher percentages of *Actinobacteria* (30.48%) and *Proteobacteria* (18.70%) in wound swabs. In addition to the dominant phyla seen in wound swabs, the wound biopsies showed a low abundance of *Deinococcus-Thermus* (4.42%) which are uncultured thermo-acidophiles adapted to highly resistant environments^18^. In both wound swabs and wound biopsies, *Fusobacteria* was relatively absent. In contrast to *L. braziliensis*^15^, we found that aerobic bacteria like *Pseudomonas* was more common in *L. donovani*-cutaneous leishmaniasis wounds compared to healthy skin. Facultative anaerobes were also detected in greater abundance than strict anaerobes in our cutaneous leishmaniasis lesions. It has been reported that the presence of facultative anaerobes in wounds act as a poor prognostic factor in the healing of cutaneous wounds^19^. Also, discrete differences, such as the presence of soil-associated bacteria (i.e., *Acidobacteria*), could be driven by environmental factors of our study population (a high degree of farming)^20^. Organisms such as *Proteus* spp., *Citrobacter* spp., *Morganella* spp. and *Propionibacterium* spp., which are common in chronic wound microbiome^21^ were relatively low/absent in these cutaneous leishmaniasis lesions suggesting that *L. donovani* associated cutaneous leishmaniasis lesions may have a distinct microenvironment in comparison with other chronic wounds.

*Actinobacteria*, *Firmicutes*, *Proteobacteria*, *Bacteroidetes* are described as the four dominant phyla of healthy human skin^22^. Similarly, these were the most common in our contralateral skin samples. We also detected a considerable percentage of *Planctomycetes* (7.46%) in contralateral skin swabs which is not commonly associated with healthy skin but has been highlighted as a potentially pathogenic organism^23^.

This study evaluated the bacterial profile of cutaneous leishmaniasis wound biopsies. In concordance with the literature on diabetic foot ulcers, the biopsy had significantly lower diversity values compared to swabs^24^. In the cutaneous leishmaniasis wound biopsies, *Staphylococcus, Pseudomonas* and *Streptococcus* which are common wound pathogens were detected^25^. *Micrococcus*, *Bacillus* spp., and *Corynebacterium* were also seen in wound biopsies which are usually present in normal skin flora, but with a potential pathogenic role if seen in deep tissues^26^. However, other common wound pathogens such as *Enterococcus spp.*, *E. coli*, *Proteus mirabilis*, *Bacteroides fragilis* were less dominant in these cutaneous leishmaniasis wounds^25^.

Through diversity analysis, we have demonstrated that cutaneous leishmaniasis lesions have significantly different alpha and beta diversities compared to contralateral and adjacent skin. Distinct profiles have previously been described in cutaneous leishmaniasis lesions caused by *L. major*^5^ and *L. braziliensis*^15^, with evidence that lesion microbiomes are transmissible to the adjacent skin in murine models^5^. Here, we did not find any significant similarity in microbiomes of the cutaneous leishmaniasis lesion and adjacent skin of 1 cm distant to the lesion. Whilst Gimblet et al., 2017 detected this transmission of lesion microbiome constituents to adjacent skin after 12 weeks post-infection^5^, our group of patients mostly (59%) had lesions of ≤ 3 months duration. Therefore, if transmission does occur it is possibly undetectable at an earlier sampling stage.

We assessed the microbiome of different types of lesions. In addition, we statistically compared the microbiomes of small and large lesions, and lesions with high and low parasite loads. We found that even very small cutaneous leishmaniasis lesions (including ulcerated papules) had well-developed biofilms. However, ulcerated papules lacked potentially pathogenic OTUs, such as genus *Corynebacterium* and *Micrococcus*, compared to wet ulcers. OTUs of *Corynebacterium* and class *Bacilli* were significantly higher in relative abundance in wound biopsies of large lesions and were also significantly higher in lesions with biofilms. Even though the size of the lesion was not significantly associated with biofilm formation, these potentially pathogenic biofilms might have influenced the lesion size. An OTU belonging to the genus of *Trabulsiella* was significantly higher in relative abundance in wound biopsies of lesions with high parasite loads. This genus can be a potentially opportunistic pathogen of faecal origin^27^.

In conclusion, our results indicate that *Leishmania donovani*-associated cutaneous leishmaniasis wounds have a distinct, less diverse, microbiome than adjacent and contralateral skin. The microbiome of the wound biopsies included common wound pathogens (*Staphylococcus*, *Pseudomonas* and *Streptococcus*) and potentially pathogenic organisms (*Micrococcus*, *Bacillus spp.*, and *Corynebacterium)*. Greater abundances of potentially pathogenic organisms were observed in wet ulcers, lesions with high parasite loads and large wounds. More than half of the wounds (61.5%) had bacterial biofilms significantly associated with an OTU belonging to class *Bacilli*. All lesions with a high pus cell count (≥ 25/low power filed), 79.2% of the wet lesions and 92.3% of the symptomatic lesions had significantly higher biofilm positivity (*p* value < 0.05).

## Methods

All methods were conducted in accordance with relevant guidelines and regulations.

### Study design

A cross-sectional study was conducted at Base Hospital Tangalle, Southern Province, Sri Lanka from July 2019 to October 2020 (cutaneous leishmaniasis is endemic to this location and the only identified species is *L. donovani*). Patients above 18 years with highly suspicious clinical ulcerated lesions, giving informed written consent were included. Exclusion criteria included: any person with debilitating illness or immunosuppression, or had previously received standard treatment for cutaneous leishmaniasis for the same presenting lesion, or recently (within the last 6 months) visited a cutaneous leishmaniasis endemic country (to exclude the possibility of including cutaneous leishmaniasis lesions caused by other species), had lesions at sites from which punch biopsies could not be taken (i.e., eyelid, pinna of the ear), received antibiotic treatment or herbal applications during the past 2 weeks^28^, or used “Lifebuoy” soap product (proven to be bactericidal and is used commonly in Sri Lanka^29^), did not have ulcerated lesions^30^, were laboratory-confirmed to have Diabetes mellitus with HbA1c level > 6.5%^31^, or patients with other skin disorders which are a major confounding factor in wound and microbiome investigations.

An interviewer-administered case report form was used to gather demographic and clinical data.

### Sample size calculation

A minimum sample size of 36 was required to estimate the proportion of cutaneous leishmaniasis lesions having a biofilm as 30% (based on preliminary work) with an alpha error of 0.05 and an acceptable difference of 0.15. A consecutive sample of 39 individuals with cutaneous leishmaniasis lesions satisfying inclusion and exclusion criteria was investigated for the presence of biofilms. The same number of patients were included in microbiome analysis as well.

### Sample collection

In addition to the below-mentioned samples, two slit skin smears, an impression smear prepared from the wound biopsy and a 2 ml venous blood sample (for high proficiency liquid chromatographic HbA1c assay to exclude Diabetes mellitus) were collected.

#### Skin swabs and wound swabs

Three swabs were collected from contralateral skin, adjacent skin and wound before cleaning^22^. The wound scab was removed before the collection of the wound swab. An adjacent skin swab was taken 1 cm away from the lesion edge^32^. Sterile COPAN Flocked swabs were pre-moistened in a solution containing 50 mM Tris, 1 mM EDTA, 0.5% Tween 20. Contralateral and adjacent skin swabs were taken following rubbing an area of 4 cm^2^, 50 times (30 s) with pressure^33^. The swab head was cut with sterile scissors and put directly into MO BIO Power soil bead tube (QIAGEN, Germany). This was transported in ice and stored in -20 °C till DNA extraction (maximum storage time was 24 h).

#### Collection of wound biopsies

A full-thickness wound biopsy (3-5 mm) from the wound edge with a catchment of the wound base was obtained from each patient under aseptic conditions following cleaning with Povidone Iodine. Aseptic conditions were maintained as obtaining a biopsy can introduce infections to the exciting wound. The wound edge was sampled instead of only the wound base because the *Leishmania* parasites aggregate at the lesion edge^9^.

Each biopsy sample was divided into three sections. One section of the wound biopsy (Section A) was transported in MO BIO Power Soil bead tube (QIAGEN, Germany), in ice and immediately transferred to -20 °C until DNA extraction (maximum storage time was 24 h). The second section (Section B) was transported in 10% Buffered Neutral Formalin^34^ and fixed for 24 h at room temperature^35^. The third section (Section C) was transported in 2.5% Glutaraldehyde with 0.1 M phosphate buffer and stored at 4^o^C^36^.

### Laboratory protocols of the study

DNA extracts from section A of the wound biopsy were used for the disease confirmation by a previously described 18S PCR with LITSR/L5.8S primers targeting the ITS1 region of Genus *Leishmania* with a sensitivity of 92.1% and specificity of 100% to detect *L. donovani*^37^.

The 2 slit skin smears were Giemsa stained and examined under oil immersion for *Leishmania* amastigotes. Parasite load calculation in these smears was done as described in the literature^38^.

DNA extracts of the swabs (4.4.1) and section A of the wound biopsy (4.4.2) were subjected to microbiome analysis by NGS (4.4.3).

Three different imaging techniques were used to visualize biofilms. Section B of the wound biopsy was histopathologically processed to make formalin-fixed paraffin-embedded tissues (FFPE) and subjected to Gram staining (Gram-FFPE, 4.4.4) and fluorescence in situ hybridization (FISH-FFPE, 4.4.5). Section C of the wound biopsy was subjected to scanning electron microscopy (SEM, 4.4.6). There is no gold standard in the detection of biofilms to date^39^. Hence, in this study, we used FISH as the reference standard in confirming biofilms of these cutaneous leishmaniasis lesions^39^ and the rest as supportive evidence. A pig skin model was prepared for biofilm controls^40^. If 2 of 3 criteria (evidence of bacterial attachment to a surface, aggregations of bacteria (microcolony formation) and presence of extracellular polymeric substances) were present, the presence of biofilm was confirmed^13^. Bacterial aggregations measuring > 5 µm were considered under the second criterion^32^.

The impression smear was gram stained to calculate the pus cell count. Pus cell count of ≥ 3 + (≥ 25 pus cells/low power field) was taken to confirm the presence of potential infection/inflammation^41^.

#### DNA extraction

Extractions were performed using DNeasy Power Soil bead tube (QIAGEN, Germany) according to manufacturer’s guidelines^42^ with modifications as suggested in the literature^43^. C1 to C6 are solutions that come with the extraction kit.

The power bead tube with the swab head was vortexed briefly before adding 60 µl of C1. The tubes were then vortexed horizontally for 15 min and incubated at 70 °C for 15 min. Following centrifuging, the supernatant was transferred to a collection tube and 250 µl of C2 was added. This was incubated at 4 °C for 5 min and centrifuged. The same procedure was repeated following the addition of 200 µl of C3. The supernatant was transferred to a new collection tube and 1200 µl of C4 was added. Following vortexing, the total volume was transferred as 675 µl aliquots to the spin column and centrifuged discarding the flow-through. 500 µl of C5 was added to the drum and centrifuged. DNA was yielded in 50 µl of C6.

#### DNA extraction from section A of the wound biopsy

This was done as described above with the flowing modifications mentioned^44^.

The biopsy tissue was chopped using sterile blades and added to the same bead tube. It was bead beat, horizontally for 1 min for homogenization of tissue and incubated at 56 °C for 20 min following the addition of C1 and 20 µl of Proteinase K (QIAGEN, Germany).

#### Illumina MiSeq amplicon sequencing

Paired-end amplicon sequencing was carried out at the Centre for Genomic Research, University of Liverpool, Crown Street, Liverpool, L69 7ZB, United Kingdom. Sequencing protocol was carried out as described in Illumina 16S library preparation guide with Illumina primers with overhang adaptor sequences targeting 16S V3 and V4 @@region^45^.

- Forward Primer: 5'TCGTCGGCAGCGTCAGATGTGTATAAGAGACAGCCTACGGGNGGCWGCAG.
- Reverse Primer: 5'GTCTCGTGGGCTCGGAGATGTGTATAAGAGACAGGACTACHVGGGTATCTAATCC.

#### FFPE-Gram (section B of the wound biopsy) to visualize bacterial biofilms

Routine Gram staining was done following deparaffinization of FFPE tissue sections. Safranin was used to stain the extra polymer matrix of bacterial biofilms^46^. Slides were examined under a bright field of an Olympus FSX100 microscope (× 40).

#### FFPE-FISH (section B of the wound biopsy) to visualize bacterial biofilms

A Cyanine3 tagged Eu-bacterial probe (EUB 338 5′Cy3-GCT GCC TCC CGT AGG AGT3′) was used to mark the bacteria (Excitation-550 nm, Emission-570 nm)^13^. The probe was synthesized at Integrated DNA Technologies, USA. Probe dilution was done in hybridization buffer (50% Formamide, 0.9 M NaCl, 20 mM TrisHCl (pH 7.6), 0.01% Sodium Dodecyl Sulfate (w/v); each 10 µl of hybridization buffer contained 50 ng of probe)^13^.

For enzymatic lysis, Lysozyme buffer was prepared (100 mM TrisHCl (pH 8), 50 mM Ethylenediaminetetraacetic acid, and 5 mg/ml Lysozyme (Sigma Aldrich)^13^.

For staining of the EPS of the biofilms, ConcanavalinA conjugated Alexa Fluor 488 (Excitation-495 nm, Emission-643 nm) (Invitrogen, USA) was used at a concentration of 1 mg/ml^13^.

The hybridization procedure was carried out as previously described with the following modifications^47^. For enzymatic lysis, Lysozyme buffer was applied (5 µl) and incubated in a humid chamber at 45 °C for 4hrs^13^. Following post hybridization washing, the air-dried sample was subjected to staining with ConcanavalinA conjugated Alexa Fluor 488 for 1 h at room temperature.

Several sections of processed tissues were examined under an Olympus FSX 100 microscope (× 40). All samples were assessed and interpreted independently by two individuals and confirmed by a third investigator. Measurement of the diameter of the largest biofilm in each image^30^ and image enhancement was done using Image J softwre (Fuji version)^48^.

#### Scanning electron microscopy to visualize bacterial biofilms

The fixed biopsy tissue was dehydrated in a series of Ethanol^36^. The tissues were imaged using a Hitachi SU6600 Analytical Variable Pressure Field Emission Scanning Electron Microscope at 5 kV after gold sputter coating of the sample for 15 s.

### Statistical analysis

#### Bioinformatics analyses

Paired-end sequencing resulted in forward and reverse FASTQ files. These were trimmed for the presence of Illumina adapter sequences using Cutadapt version 1.2.1^49^. The option -O 3 was used, so the 3' end of any reads which match the adapter sequence for 3 bp. or more were trimmed. The reads were further trimmed using Sickle version 1.200 with a minimum window quality score of 20. Reads shorter than 15 bp after trimming were removed^50^. Quality filtering and downstream sequence analysis were performed using Qiime 2–2020.2 pipeline according to the Standard Operating Procedure (SOP) available on https://docs.qiime2.org/2020.6/tutorials/moving-pictures/^51^. The OTUs were clustered at 97% sequence similarity and taxonomy was applied by a Naïve Bayes classifier trained on this dataset using reference sequences from the latest Greengenes database (v13_8)^52^. Alpha diversity was calculated in QIIME2, statistical testing and plotting of figures were performed using GraphPad Prism version 9.2.0 for macOS, GraphPad Software, San Diego, California USA, www.graphpad.com. Beta diversity results was imported from QIIME2 and plotted using QIIME2R^53^, phyloseq^54^, tidyverse^55^ and ggplot2^56^. DESeq2 testing was performed​, with the calculation of geometric means prior to estimation of size factors, in the same software, using the DESeq2 package^57^, QIIME2R^53^, phyloseq^54^ and ggplot2^56^.

#### Data analysis of biofilm investigation

Statistical analysis of the biofilm investigation was done using R Version 4.0.1. Chi-square test with Yates' continuity correction and Fisher's Exact Test were used to test for significant associations. In all cases, biofilm positivity was confirmed by three independent reviewers. Age of the participants was grouped as < 40 and > 40 years. CL lesions (ulcers, ulcerated nodules, ulcerated papules and ulcerated plaques) were categorized as wet or dry lesions. Wet lesions had pus/sero-purulent discharge. The maximum diameter of each lesion was categorized as < 2 cm and > 2cm^58^. All the lesions were categorized as < 3 months or > 3 months of duration at the time of presentation^59^. CL lesions are usually asymptomatic; lesions with pain and/or itching at the time of presentation were categorized as symptomatic lesions. Parasite load as assessed by Giemsa-stained slit skin smears were categorized into 2 groups; lesions with a parasite load of nil or 1 + were grouped as “low parasite load” and lesions with a parasite load of 2 + to 6 + were grouped as “high parasite load”^60^. The size of the largest biofilm^61^ in each lesion was categorized as 5–10 μm, 11–50 μm and > 50 μm^62^.

### Ethical approval

Ethics approval was obtained from the Ethics Review Committee, Faculty of Medical Sciences, University of Sri Jayewardenepura, Sri Lanka (number 27/19).

## Supplementary Information

Supplementary Information.

## Data Availability

http://www.ncbi.nlm.nih.gov/bioproject/781195 (BioProject ID PRJNA781195).
