# Supplementary material for: Distinct microbiome profiles and biofilms in Leishmania donovani-driven cutaneous leishmaniasis wounds
Source: Sci Rep. 2021 Nov 30;11:23181. doi: 10.1038/s41598-021-02388-8 (PMC8633208; doi:10.1038/s41598-021-02388-8)
Supplement: Supplementary file 1 — Supplementary Information. [file 41598_2021_2388_MOESM1_ESM.docx]

**Distinct Microbiome Profiles and Biofilms in *Leishmania donovani*-Driven Cutaneous Leishmaniasis Wounds**

T D Jayasena Kaluarachchi^1*^, Paul M Campbell^2^, Rajitha Wickremasinghe^3^, Shalindra Ranasinghe^1^, Renu Wickremasinghe^1^, Surangi Yasawardene^4^, Hiromel De Silva^5^, Chandrani Menike^1^, M C K Jayarathne^6^, Subodha Jayathilake^7^, Ayomi Dilhari^8^,

Andrew J McBain^2^ and Manjula Weerasekera^9^.

1. Department of Parasitology, Faculty of Medical Sciences, University of Sri Jayewardenepura, Gangodawila, Nugegoda, Sri Lanka.

2. Division of Pharmacy and Optometry, School of Health Sciences, Faculty of Biology, Medicine and Health, The University of Manchester, United Kingdom.

3. Department of Public Health, Faculty of Medicine, University of Kelaniya, Sri Lanka.

4. Department of Anatomy, Faculty of Medical Sciences, University of Sri Jayewardenepura, Gangodawila, Nugegoda, Sri Lanka.

5. Dermatology Unit, Base Hospital, Tangalle, Sri Lanka.

6. Department of Family Medicine, Faculty of Medical Sciences, University of Sri Jayewardenepura, Gangodawila, Nugegoda, Sri Lanka.

7. Department of Pathology, Faculty of Medical Sciences, University of Sri Jayewardenepura, Gangodawila, Nugegoda, Sri Lanka.

8. Department of Basic Sciences, Faculty of Allied Health Sciences, University of Sri Jayewardenepura, Gangodawila, Nugegoda, Sri Lanka.

9. Department of Microbiology, Faculty of Medical Sciences, University of Sri Jayewardenepura, Gangodawila, Nugegoda, Sri Lanka.

*Correspondence to dilhara@sjp.ac.lk


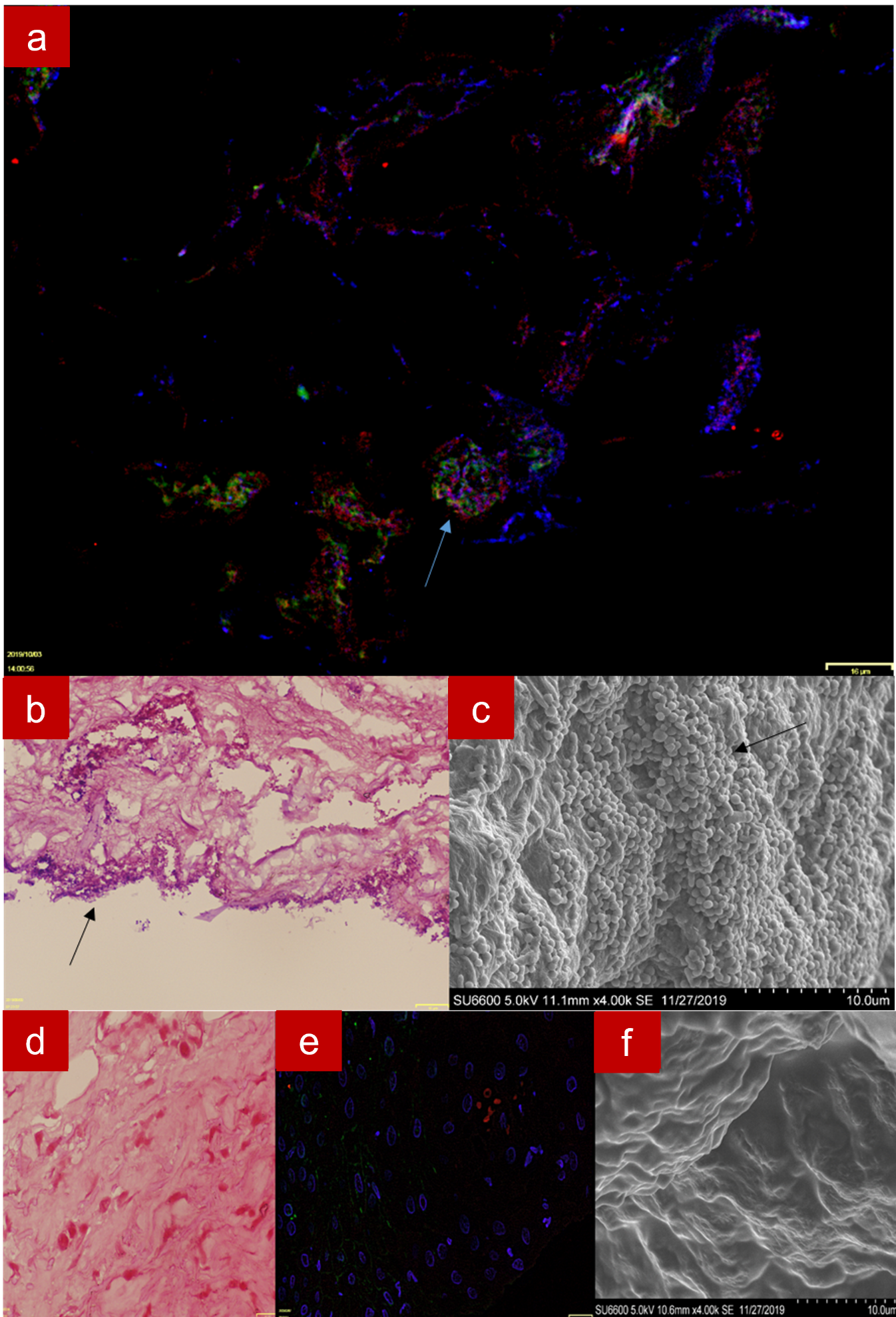


Supplementary figure 1: Results of positive and negative controls of the porcine skin biofilm model. Positive control (Arrows point at the formed biofilms): stained with fluorescence *in situ* hybridization, Bacteria seen as red dots with the CY3 tagged Eu Bacterial probe, tissue nuclei and bacterial nuclear material seen in blue with DAPI staining and extra polymeric matrix in green with Concavalin A conjugated Alexa Fluor 488 (a), stained with Gram staining (b) and imaged by scanning electron microscopy (c); Negative control: stained with Gram staining (d), fluorescence *in situ* hybridization (e) and imaged by scanning electron microscopy (f).


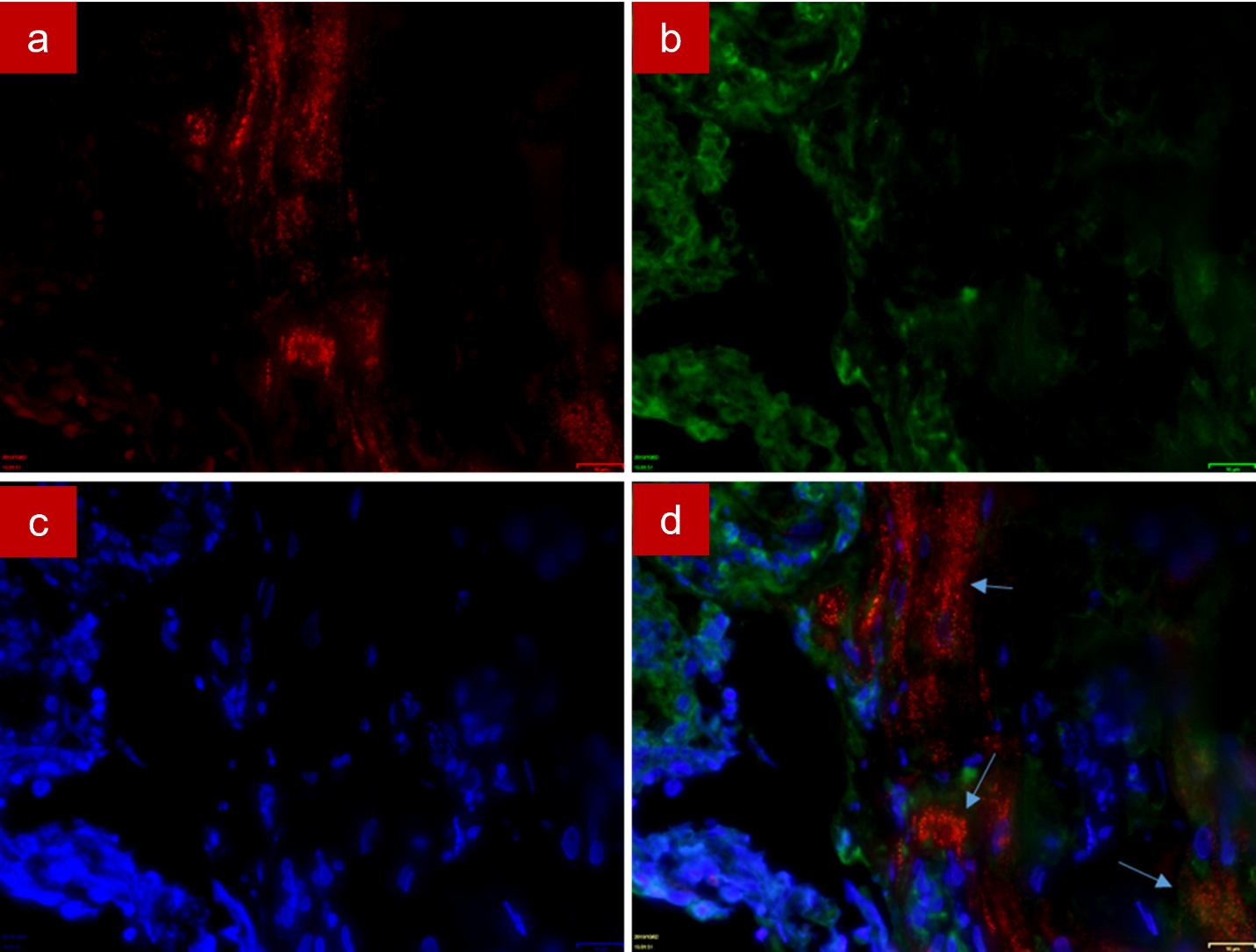


Supplementary Figure 2: Visualization of biofilms in cutaneous leishmaniasis lesions by fluorescence *in situ* hybridization. The red signal by CY 3 tagged Eu bacterial probe marking bacteria (a); The green signal by Concavalin A conjugated Alexa Fluor 488 marking the extracellular polymeric substances (b); The blue signal with DAPI marking tissue nuclei (c); The overlaid image (arrows indicating the biofilms) (d).


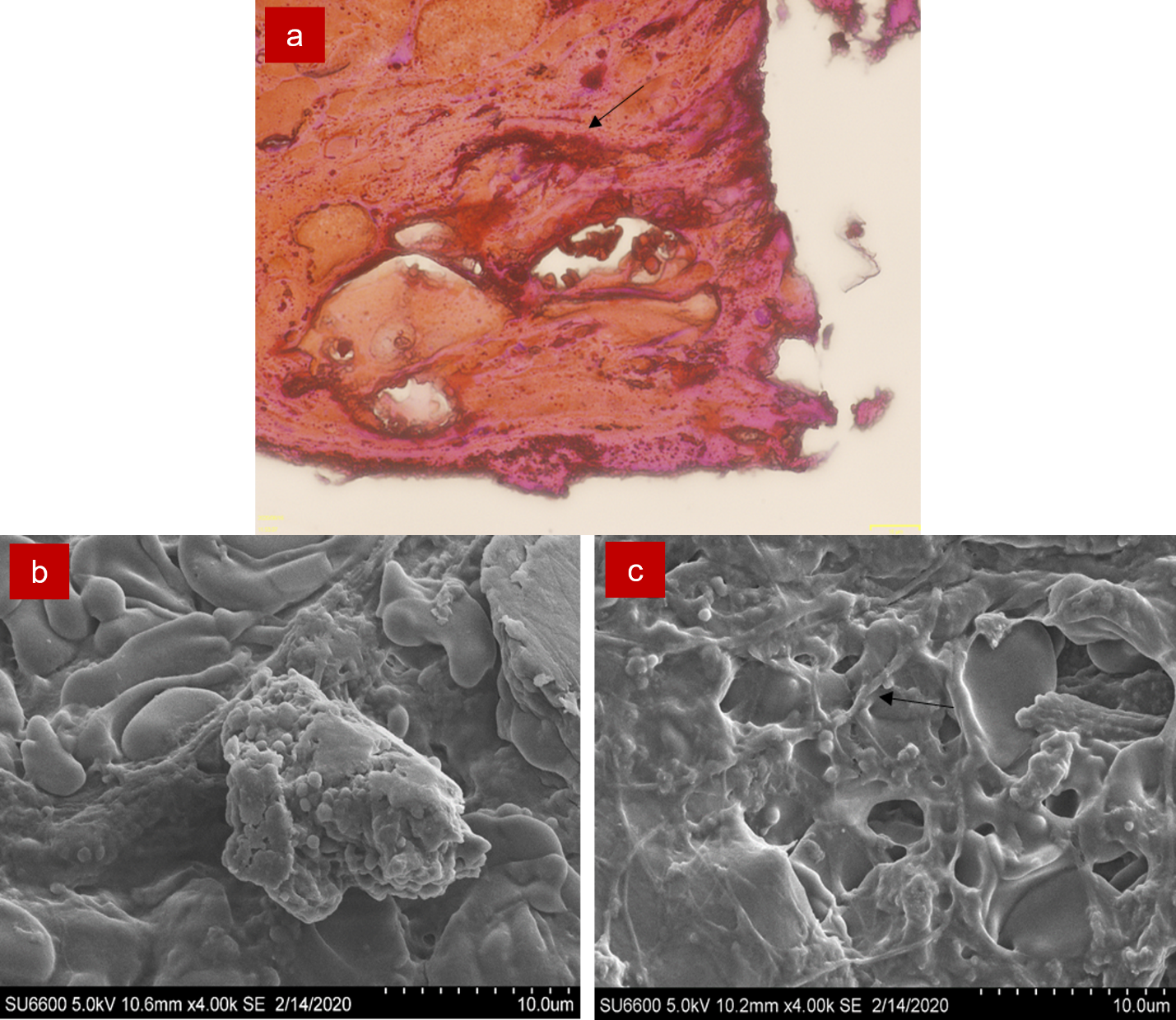


Supplementary Figure 3: Gram-positive cocci bacterial aggregates in a biofilm in a cutaneous leishmaniasis lesion. Extracellular polymeric substances seen in orange (a). Scanning electron microscopy images of coccoid cells, with smooth extracellular polymeric substances (b); thin thread-like extracellular polymeric substance (c).

Supplementary Table 1: DESeq2 analysis results showing significantly different OTUs between each sample type (p.adj<0.05).

|  | OTUs | Phylum | Class | Order | Family | Genus | Species |  |  |
| --- | --- | --- | --- | --- | --- | --- | --- | --- | --- |
| CS vs AS |  |  |  |  |  |  |  | CS | AS |
|  | OTU1 | Planctomycetes | Planctomycetia | Planctomycetales | Planctomycetaceae | Planctomyces | NA | 0.00% | 0.03% |
|  | OTU2 | Actinobacteria | Actinobacteria | Actinomycetales | Micrococcaceae | Micrococcus | NA | 0.11% | 0.04% |
| WS vs CS |  |  |  |  |  |  |  | WS | CS |
|  | OTU3 | Proteobacteria | Gammaproteobacteria | Pseudomonadales | Moraxellaceae | Acinetobacter | NA | 0.00% | 0.07% |
|  | OTU4 | Proteobacteria | Deltaproteobacteria | Myxococcales | Cystobacteraceae | Cystobacter | NA | 0.00% | 0.05% |
|  | OTU5 | Actinobacteria | Actinobacteria | Actinomycetales | Dermatophilaceae | Piscicoccus | intestinalis | 0.00% | 0.03% |
|  | OTU6 | Planctomycetes | Planctomycetia | Gemmatales | Isosphaeraceae | NA | NA | 0.00% | 0.04% |
|  | OTU7 | Planctomycetes | Planctomycetia | Planctomycetales | Planctomycetaceae | Planctomyces | NA | 0.00% | 0.03% |
|  | OTU8 | Cyanobacteria | Chloroplast | Streptophyta | NA | NA | NA | 0.00% | 0.06% |
|  | OTU9 | Planctomycetes | Planctomycetia | Gemmatales | Gemmataceae | NA | NA | 0.01% | 0.04% |
|  | OTU10 | Firmicutes | Bacilli | NA | NA | NA | NA | 20.51% | 0.10% |
|  | OTU11 | Firmicutes | Bacilli | Bacillales | Planococcaceae | NA | NA | 0.00% | 0.03% |
|  | OTU12 | Proteobacteria | Gammaproteobacteria | Pseudomonadales | Pseudomonadaceae | NA | NA | 0.00% | 0.03% |
|  | OTU13 | Actinobacteria | Actinobacteria | Actinomycetales | Nocardioidaceae | Nocardioides | NA | 0.02% | 0.04% |
|  | OTU14 | Proteobacteria | Gammaproteobacteria | Enterobacteriales | Enterobacteriaceae | NA | NA | 0.05% | 0.03% |
|  | OTU15 | Firmicutes | Bacilli | Bacillales | Staphylococcaceae | Staphylococcus | sciuri | 0.05% | 0.04% |
|  | OTU16 | Proteobacteria | Gammaproteobacteria | Aeromonadales | Aeromonadaceae | NA | NA | 0.03% | 0.04% |
|  | OTU17 | Actinobacteria | Actinobacteria | Actinomycetales | Nocardioidaceae | NA | NA | 0.01% | 0.04% |
|  | OTU18 | Cyanobacteria | Oscillatoriophycideae | Chroococcales | Xenococcaceae | NA | NA | 0.00% | 0.03% |
|  | OTU19 | Proteobacteria | Alphaproteobacteria | Rhizobiales | Bradyrhizobiaceae | Balneimonas | NA | 0.00% | 0.03% |
|  | OTU20 | Firmicutes | Bacilli | Lactobacillales | Streptococcaceae | Streptococcus | NA | 0.52% | 0.04% |
| WS vs AS |  |  |  |  |  |  |  | WS | AS |
|  | OTU8 | Cyanobacteria | Chloroplast | Streptophyta | NA | NA | NA | 0.00% | 0.06% |
|  | OTU21 | Bacteroidetes | Flavobacteriia | Flavobacteriales | [Weeksellaceae] | Cloacibacterium | NA | 0.01% | 0.05% |
|  | OTU22 | Planctomycetes | Planctomycetia | Gemmatales | Isosphaeraceae | NA | NA | 0.01% | 0.05% |
|  | OTU11 | Firmicutes | Bacilli | Bacillales | Planococcaceae | NA | NA | 0.00% | 0.07% |
|  | OTU23 | Acidobacteria | [Chloracidobacteria] | RB41 | Ellin6075 | NA | NA | 1.24% | 0.07% |
| WS vs WB |  |  |  |  |  |  |  | WS | WB |
|  | OTU24 | Firmicutes | Bacilli | Bacillales | Bacillaceae | Marinococcus | NA | 0.50% | 0.00% |
|  | OTU25 | Firmicutes | Bacilli | Bacillales | Planococcaceae | Staphylococcus | saprophyticus | 1.32% | 0.00% |
|  | OTU26 | Actinobacteria | Actinobacteria | Actinomycetales | Micrococcaceae | Nesterenkonia | NA | 0.20% | 0.00% |
|  | OTU27 | Actinobacteria | Actinobacteria | Actinomycetales | Pseudonocardiaceae | Saccharopolyspora | NA | 0.20% | 0.07% |
|  | OTU28 | Actinobacteria | Actinobacteria | Actinomycetales | Dietziaceae | NA | NA | 0.15% | 0.00% |
|  | OTU29 | Actinobacteria | Actinobacteria | Actinomycetales | Micrococcaceae | NA | NA | 0.43% | 0.00% |
|  | OTU30 | Firmicutes | Bacilli | Gemellales | NA | NA | NA | 0.47% | 0.00% |
|  | OTU31 | Firmicutes | Clostridia | Clostridiales | Veillonellaceae | Veillonella | NA | 0.08% | 0.00% |
|  | OTU32 | Actinobacteria | Actinobacteria | Actinomycetales | Pseudonocardiaceae | Actinomycetospora | NA | 0.05% | 0.00% |
|  | OTU33 | Proteobacteria | Gammaproteobacteria | Xanthomonadales | Xanthomonadaceae | NA | NA | 0.19% | 0.18% |
|  | OTU34 | Actinobacteria | Actinobacteria | Actinomycetales | Micrococcaceae | Nesterenkonia | NA | 0.06% | 0.00% |
|  | OTU35 | Proteobacteria | Gammaproteobacteria | Xanthomonadales | Xanthomonadaceae | NA | NA | 0.08% | 0.00% |
|  | OTU36 | Actinobacteria | Actinobacteria | Actinomycetales | Corynebacteriaceae | Corynebacterium | NA | 0.07% | 0.00% |
|  | OTU37 | Actinobacteria | Actinobacteria | Actinomycetales | Corynebacteriaceae | Corynebacterium | NA | 0.06% | 0.00% |
|  | OTU38 | Firmicutes | Bacilli | Lactobacillales | Carnobacteriaceae | Granulicatella | NA | 0.12% | 0.00% |
|  | OTU39 | Cyanobacteria | Chloroplast | Streptophyta | NA | NA | NA | 0.10% | 0.00% |
|  | OTU40 | Firmicutes | Bacilli | Bacillales | Staphylococcaceae | Staphylococcus | NA | 0.32% | 0.09% |
|  | OTU41 | Actinobacteria | Actinobacteria | Actinomycetales | Corynebacteriaceae | Corynebacterium | NA | 0.12% | 0.00% |
|  | OTU42 | Firmicutes | Bacilli | Gemellales | NA | NA | NA | 0.09% | 0.01% |
|  | OTU20 | Firmicutes | Bacilli | Lactobacillales | Streptococcaceae | Streptococcus | NA | 0.52% | 0.57% |
|  | OTU43 | Firmicutes | Bacilli | Lactobacillales | Streptococcaceae | Streptococcus | NA | 0.67% | 0.00% |
|  | OTU44 | Actinobacteria | Actinobacteria | Actinomycetales | Intrasporangiaceae | NA | NA | 1.19% | 0.04% |
|  | OTU45 | Actinobacteria | Actinobacteria | Actinomycetales | Dermabacteraceae | Brachybacterium | NA | 1.66% | 0.27% |
|  | OTU46 | Actinobacteria | Rubrobacteria | Rubrobacterales | Rubrobacteraceae | Rubrobacter | NA | 0.25% | 0.00% |
|  | OTU47 | Proteobacteria | Alphaproteobacteria | Rhodobacterales | Rhodobacteraceae | Paracoccus | NA | 0.20% | 0.01% |
|  | OTU48 | Firmicutes | Bacilli | Lactobacillales | Streptococcaceae | Streptococcus | NA | 0.14% | 0.02% |
|  | OTU49 | Proteobacteria | Gammaproteobacteria | Pseudomonadales | Pseudomonadaceae | Pseudomonas | NA | 2.99% | 2.39% |
|  | OTU23 | Acidobacteria | [Chloracidobacteria] | RB41 | Ellin6075 | NA | NA | 1.24% | 21.02% |
|  | OTU50 | Actinobacteria | Actinobacteria | Actinomycetales | Brevibacteriaceae | Brevibacterium | NA | 0.11% | 0.00% |
|  | OTU51 | Proteobacteria | Alphaproteobacteria | Rhodobacterales | Rhodobacteraceae | Paracoccus | NA | 0.62% | 0.03% |
|  | OTU52 | Proteobacteria | Alphaproteobacteria | Sphingomonadales | Sphingomonadaceae | NA | NA | 0.08% | 0.00% |
|  | OTU53 | Actinobacteria | Actinobacteria | Actinomycetales | Micrococcaceae | Kocuria | NA | 0.19% | 0.00% |
|  | OTU54 | Actinobacteria | Actinobacteria | Actinomycetales | Corynebacteriaceae | Corynebacterium | NA | 0.04% | 0.00% |
|  | OTU55 | Actinobacteria | Actinobacteria | Actinomycetales | Brevibacteriaceae | Brevibacterium | aureum | 0.13% | 0.00% |
|  | OTU56 | Bacteroidetes | Flavobacteriia | Flavobacteriales | [Weeksellaceae] | Chryseobacterium | NA | 0.32% | 0.09% |
|  | OTU57 | Proteobacteria | Gammaproteobacteria | Pseudomonadales | Moraxellaceae | Enhydrobacter | NA | 1.94% | 2.32% |
|  | OTU58 | Actinobacteria | Actinobacteria | Actinomycetales | Pseudonocardiaceae | Saccharopolyspora | NA | 0.16% | 0.40% |
|  | OTU59 | Proteobacteria | Gammaproteobacteria | Pseudomonadales | Moraxellaceae | Acinetobacter | NA | 0.33% | 0.16% |
|  | OTU60 | Firmicutes | Clostridia | Clostridiales | Veillonellaceae | Veillonella | dispar | 0.57% | 0.00% |
|  | OTU61 | Actinobacteria | Rubrobacteria | Rubrobacterales | Rubrobacteraceae | Rubrobacter | NA | 0.03% | 1.80% |
|  | OTU62 | Firmicutes | Clostridia | Clostridiales | Veillonellaceae | Veillonella | parvula | 0.08% | 0.00% |
|  | OTU63 | Actinobacteria | Actinobacteria | Actinomycetales | Corynebacteriaceae | Corynebacterium | NA | 0.93% | 0.04% |
|  | OTU64 | Proteobacteria | Gammaproteobacteria | Pasteurellales | Pasteurellaceae | Haemophilus | parainfluenzae | 0.11% | 0.00% |
|  | OTU65 | Actinobacteria | Rubrobacteria | Rubrobacterales | Rubrobacteraceae | Rubrobacter | NA | 0.09% | 2.71% |
|  | OTU66 | Firmicutes | Clostridia | Clostridiales | [Tissierellaceae] | Finegoldia | NA | 0.06% | 0.00% |
|  | OTU67 | Actinobacteria | Actinobacteria | Actinomycetales | Dermacoccaceae | Dermacoccus | NA | 0.12% | 0.00% |
|  | OTU68 | Actinobacteria | Actinobacteria | Actinomycetales | Corynebacteriaceae | Corynebacterium | NA | 0.86% | 0.12% |
|  | OTU69 | Thermus | Deinococci | Thermales | Thermaceae | Meiothermus | NA | 0.10% | 2.96% |
|  | OTU70 | Acidobacteria | [Chloracidobacteria] | RB41 | Ellin6075 | NA | NA | 0.01% | 0.17% |
|  | OTU71 | Actinobacteria | Actinobacteria | Actinomycetales | Corynebacteriaceae | Corynebacterium | NA | 0.14% | 0.00% |

CS: Contralateral skin swab; AS: Adjacent skin swab; WS: Wound swab; WB: Wound biopsy; NA: not applicable

Supplementary Table 2: Clinico-demographic characteristics by presence or absence of biofilm.

| **Demographic/ Clinical parameter** |  | **Characteristic** | | **P-value** |
| --- | --- | --- | --- | --- |
| **Gender** |  | **Male** | **Female** |  |
|  | **Number of cases N (%)** | 21 (53.8%) | 18 (46.2%) |  |
|  | **Biofilm positive N (%)** | 14 (58.3%) | 10 (41.7%) | P_1_=0.703 |
|  | **Biofilm negative N (%)** | 7 (46.7%) | 8 (53.3%) |  |
| **Age** |  | **<40 years** | **>40years** |  |
|  | **Number of cases N (%)** | 12 (30.8%) | 27 (69.2%) |  |
|  | **Biofilm positive N (%)** | 4 (16.7%) | 20 (83.3%) | P_2_=0.031 |
|  | **Biofilm negative N (%)** | 8 (53.3%) | 7 (46.7%) |  |
| **Type of lesion** |  | **Wet lesions** | **Dry lesions** |  |
|  | **Number of cases N (%)** | 23 (59.0%) | 16 (41.0%) |  |
|  | **Biofilm positive N (%)** | 19 (79.2%) | 5 (20.8%) | P_1_=0.004 |
|  | **Biofilm negative N (%)** | 4 (26.7%) | 11(73.3) |  |
| **Size of lesions** |  | **<2cm** | **>2cm** |  |
|  | **Number of cases N (%)** | 19 (48.7%) | 20(51.3%) |  |
|  | **Biofilm positive N (%)** | 12 (50.0%) | 12 (50.0%) | P_1_=1.000 |
|  | **Biofilm negative N (%)** | 7 (46.7%) | 8 (53.3%) |  |
| **Location of the lesion** |  | **Upper limb** | **Lower limb** |  |
|  | **Number of cases N (%)** | 23 (59.0%) | 16 (41.0%) |  |
|  | **Biofilm positive N (%)** | 15 (62.5%) | 9 (37.5%) | P_1_=0.817 |
|  | **Biofilm negative N (%)** | 8 (53.3%) | 7 (46.7%) |  |
| **Duration of the lesion at time of presentation** |  | **<3months** | **>3months** |  |
|  | **Number of cases N (%)** | 23 (59.0%) | 16 (41.0%) |  |
|  | **Biofilm positive N (%)** | 17 (70.8%) | 7 (29.2%) | P_2_=0.386 |
|  | **Biofilm negative N (%)** | 6 (40.0%) | 9 (60.0%) |  |
| **Symptoms** |  | **Asymptomatic** | **Symptomatic** |  |
|  | **Number of cases N (%)** | 26 (66.7%) | 13(33.3%) |  |
|  | **Biofilm positive N (%)** | 12 (50.0%) | 12 (50.0%) | P_1_=0.015 |
|  | **Biofilm negative N (%)** | 14(93.3%) | 1 (6.7%) |  |
| **Parasite load in lesions** |  | **Low** | **High** |  |
|  | **Number of cases N (%)** | 17 (43.6%) | 22 (56.4%) |  |
|  | **Biofilm positive N (%)** | 9 (37.5%) | 15 (62.5%) | P_1_=0.523 |
|  | **Biofilm negative N (%)** | 8 (53.3%) | 7 (46.7%) |  |
| **Pus cell count** |  | **< 3+** | **> 3+** |  |
|  | **Number of cases N (%)** | 30 (76.9%) | 9 (23.1%) |  |
|  | **Biofilm positive N (%)** | 15 (62.5%) | 9 (37.5%) | P_2_=0.007 |
|  | **Biofilm negative N (%)** | 15 (100%) | 0 (0%) |  |

P_1_: p value of Pearson's Chi-squared test with Yates' continuity correction; P_2_: p value of Fisher's Exact test
